# Cognitive load promotes honesty

**DOI:** 10.1007/s00426-022-01686-8

**Published:** 2022-06-01

**Authors:** Moritz Reis, Roland Pfister, Anna Foerster

**Affiliations:** Department of Psychology (III), University of Würzburg, Röntgenring 11, 97070 Würzburg, Germany

## Abstract

In three experiments, we examined the cognitive underpinnings of self-serving dishonesty by manipulating cognitive load under different incentive structures. Participants could increase a financial bonus by misreporting outcomes of private die rolls without any risk of detection. At the same time, they had to remember letter strings of varying length. If honesty is the automatic response tendency and dishonesty is cognitively demanding, lying behavior should be less evident under high cognitive load. This hypothesis was supported by the outcome of two out of three experiments. We further manipulated whether all trials or only one random trial determined payoff to modulate reward adaptation over time (Experiment 2) and whether payoff was framed as a financial gain or loss (Experiment 3). The payoff scheme of one random or all trials did not affect lying behavior and, discordant to earlier research, facing losses instead of gains did not increase lying behavior. Finally, cognitive load and incentive frame interacted significantly, but contrary to our assumption gains increased lying under low cognitive load. While the impact of cognitive load on dishonesty appears to be comparably robust, motivational influences seem to be more elusive than commonly assumed in current theorizing.

Individual dishonesty poses a great threat to the functioning of modern societies. Whether it is compliance with COVID-19 rules or tax returns, authorities have to trust citizens to be honest in many circumstances because available resources or ethical considerations do not allow for close supervision of such everyday behavior. Against this background, a thorough understanding of the factors that promote or reduce dishonest actions appears vital. Despite considerable efforts, however, key questions on the motivational and cognitive underpinnings of dishonest behavior remain controversial. This is especially true for the question of whether decision-making and action execution are more geared towards honesty or towards dishonesty. In three experiments, we therefore investigated whether cognitive load modulates the emergence of dishonesty and how this modulation is affected by different incentive characteristics. Moreover, we closely investigated the temporal development of lying behavior for different incentive structures.

A whole body of research examined whether lies are the result of an automatic tendency to serve self-interest or whether they instead rely on demanding cognitive processes. This dichotomy of automatic and deliberative decision-making has been applied to numerous areas in different academic disciplines (Gawronski & Creighton, 2013). Research on dishonesty is yet to come to a clear conclusion, however. Matters are especially complex in this field because different measures have yielded opposing patterns. Performance measures, focusing on how dishonest actions are cognitively controlled, have yielded strong evidence for direct retrieval of honest rather than dishonest action tendencies (Debey et al., 2014; Duran et al., 2010; Foerster et al., 2017, 2019; Furedy et al., 1988; Nuñez et al., 2005; Spence et al., 2001). Many of these studies deliberately focused on instructed lies without additional motivational incentives for dishonesty. Studies that addressed self-serving lies in the face of tempting rewards, by contrast, typically assessed choice behavior rather than performance. One exemplary setup in this research tradition is the *die under the cup paradigm* (Fischbacher & Föllmi-Heusi, 2013) that will also be used in the present study. Participants usually report the outcome of a covert die roll whereby higher reports result in higher payoffs. Consequently, lying is self-serving with no chance of detection in this setup. Participants therefore claim higher payoffs than expected by chance, which indicates that lying takes place at least to some degree (Foerster et al., 2013; Hilbig & Hessler, 2013; Schindler & Pfattheicher, 2017).

Even though such measures cannot be directly informative for the question of which decision and action tendencies emerge during dishonesty (Pfister, 2022), it is still notable that studies on incentivized, unsolicited lying often found a consistent trend towards dishonesty (Fischbacher & Föllmi-Heusi, 2013; Gneezy et al., 2018; Mazar et al., 2008). Some researchers have, therefore, interpreted such findings as evidence for dishonesty as the automatic response provided there is an incentive to lie (see, e.g., Bereby-Meyer & Shalvi, 2015; Köbis et al., 2019). Evaluating this claim requires elaborate experimental designs that overcome the inherently limited temporal resolution of choice behavior as a main measure, however (Lohse et al., 2018). One possibility to overcome this limitation is to assess the impact of time pressure on the frequency of dishonest responding, with evidence pointing towards more honesty under time pressure (Capraro, 2017; Capraro et al., 2019; see also Foerster et al., 2013; Van der Cruyssen et al., 2020).

The present experiments aimed at providing converging evidence using cognitive load to probe for differences in automaticity between honest and dishonest responding. Assuming that lying behavior is based on cognitively demanding processes, we propose that limiting available cognitive resources by a second task should directly affect dishonesty (Sporer, 2016). This proposal has been explored thoroughly in the field of lie detection (Vrij et al., 2017; Walczyk et al., 2013), and we aimed to study whether corresponding findings would also translate to the outcome of motivated decisions in the face of a tempting option. Indeed, there is broad evidence from different research fields that high cognitive load favors impulsive and automatic behavior over controlled actions (for an overview on the effect of cognitive load on decision-making, see Deck & Jahedi, 2015). For instance, high cognitive load led to less strategical behavior in economical decision-making games (e.g., Duffy & Smith, 2014) and increased use of recently activated stereotypes (e.g., Gilbert & Hixon, 1991). In the field of dishonesty research, a first between-subject study suggests that high cognitive load decreases self-serving dishonesty significantly (Van’t Veer et al., 2014). Participants reported the outcomes of three consecutive die rolls, whereby only the first outcome determined payoff. The authors varied cognitive load in a between-subjects design by presenting letter strings of different lengths (two or seven letters) that participants had to remember while completing the *die under the cup task*. Significantly higher and, therefore, more dishonest outcomes were reported in the low cognitive load than in the high cognitive load condition.

The current study builds on these results and aims at an integrated investigation of both, the cognitive architecture and motivational preconditions of dishonesty in three experiments. Even though these research questions have been targeted by earlier studies, the state of research is not consistent (e.g., Köbis et al., 2019) and several basic findings could not be replicated (e.g., Kristal et al., 2020; Van der Cruyssen et al., 2020; Verschuere et al., 2018). Accordingly, a larger database and refined empirical approaches are necessary to answer central questions about how lies come about. The present studies, therefore, not only aimed to test the replicability of earlier findings concerning the effect of cognitive load on dishonesty (Van’t Veer et al., 2014), but especially, to investigate whether the proposed effect holds true when manipulating further situational aspects such as the incentive structure of the experimental setting. These investigations, therefore, allow to examine the generalizability and accordingly the real-world implications of experimental findings from dishonesty research (e.g., for lie detection).

To maximize information gain, our experimental setup deviated in multiple characteristics from earlier studies. In Experiment 1, we employed a more fine-grained manipulation of cognitive load in the die under the cup task than previous research (Van’t Veer et al., 2014). We further investigated different operationalizations of incentive structures to probe for eventual generalizability in Experiment 2 (reward based on one random outcome vs. all reported outcomes) and in Experiment 3 (gaining reward vs. preventing loss). Finally, in contrast to most earlier research, we did not use a between-subject, single shot design, but applied a within-subject design that did not only come with the advantage of higher statistical power but also allowed us to investigate the development of lying over time.

## Study 1

Experiment 1 investigated the extent of self-serving dishonesty under three different levels of cognitive load. We manipulated cognitive load via the length of letter strings and measured it via memory performance by asking participants to reproduce one randomly chosen letter. Following the findings of prior research in this field (e.g., Capraro, 2017; Foerster et al., 2013; Van’t Veer et al., 2014), we argue that if dishonesty requires cognitive capacity, it should decrease with increasing cognitive load. If our results support this assumption, we further hypothesized that lying should only be observable if cognitive capacity for these processes is available. That is, dishonesty should only emerge if memory performance is still better than chance.

### Method

#### Participants and design

This study was preregistered (https://osf.io/b4v2e). Due to a lack of comparable prior studies, we decided upon a sample size of 40 participants^1^ (31 females; 37 right handers; age: *M* = 29 years, SD = 10 years) who earned four euros for their participation plus a bonus of two euros (see below). We employed a within-subjects design so that each participant went through a random sequence of three letter string lengths.

#### Procedure and materials

Participants used the letter keys *D*, *F* and *G* as well as the number keys from *1* to *6* of a typical German QWERTZ keyboard to report memorized letters and outcomes of die rolls, respectively. For the die task, regular plastic cups lined with sponge rubber were used to attenuate sounds as we collected data of multiple participants in parallel. Inside each cup, there was a regular six-sided die and the cup was covered with transparent plastic foil to keep the die within the cup at all times. Letter strings of the secondary task consisted of either two, five, or eight lowercase letters. Letters instead of numbers were used to avoid interference with the numbers of the die. Every letter, independent of the length of the letter string, could be either *d*, *f*, or *g*, selected at random.

Between two and four participants performed the experiment at the same time at separated workstations in a laboratory. The experimenter also sat behind a partitioner in the same room. In the informed consent, the experiment was described as a memory experiment which allegedly examines the influence of a motoric action (shaking the cup with the die inside) on memory performance. We debriefed participants fully at the end of the study. After signing the informed consent, participants entered their age, gender and handedness on the computer. Each participant started the experiment individually on the computer, introduced by further instructions about the task.

Participants learned that they could earn additional payment depending on their performance in the task. We told participants that the bonus of one trial would be as high as the reported die roll outcome in eurocents and that they would receive the total sum of all trials at the end of the experiment together with the general payment for their participation. Therefore, participants had an incentive to lie by reporting higher numbers. In order to receive the monetary bonus for an individual reported die roll outcome, however, they also had to remember the correct letter of that trial. As such, we motivated participants to perform the memory task properly for a successful cognitive load manipulation.

Each trial began with the presentation of a fixation cross for 1500 milliseconds (ms) followed by the presentation of a letter string for 3000 ms. The letter string automatically disappeared and the participant was asked to shake the cup and report the outcome of the die roll by pressing the corresponding number on the keyboard. There was no time limit for doing so but the required time was measured for secondary analyses. After providing their die roll outcome, participants were asked to report one specific letter of the string, e.g., the second letter, by pressing the corresponding letter key on the keyboard and there was also no time limit for this task. Again, the required time was recorded, and participants received direct feedback if their answer was correct with their current bonus score.

Each participant conducted nine practice trials followed by ninety experimental trials with a randomized order of letter string lengths. A self-paced break was suggested by the computer program after half of the experimental trials. At the end, each participant had to answer two open-ended questions regarding the perceived subject of the study and whether they made unusual observations. Moreover, they were informed that each participant actually received the same final bonus of two euros to not promote lying behavior.

### Results

#### Data treatment and analyses

Raw data, the analysis syntax and programming files are available on the Open Science Framework (OSF repository: https://osf.io/bp8vt/). We did not analyze practice trials. For all dependent variables, we calculated a repeated-measures analysis of variance (rmANOVA) with the factor string length (two, five or eight letters). Greenhouse–Geisser corrections were applied if Mauchly’s test indicated a violation of the sphericity assumption.

To check whether remembering more letters was indeed more cognitively demanding, we analyzed memory performance as quantified by the number of correctly remembered letters divided by the number of all presented letter strings. As an additional, exploratory manipulation check, we analyzed the time for shaking the die and reporting the outcome (i.e., die roll and report time) as well as the time for recollecting the letter string (i.e., recollection time). In both analyses, we used medians instead of means due to an expected high variance in the sample as there was no response deadline.

We investigated our main hypothesis that cognitive load would decrease dishonesty by analyzing the average reported outcome of die rolls across string lengths to be retained in memory. We further tested the assumption that dishonesty should only be observable if enough cognitive resources are available by means of two-tailed one-sample *t*-tests for each letter string length, by which we compared memory performance to the probability of randomly guessing the correct letter (0.33) and average reported outcomes to the expected value (3.5). We planned to conduct these analyses only in case the respective rmANOVAs returned significant main effects.

To exploratively investigate the temporal development of dishonesty, we compared reported outcomes as well as memory performance between both halves of the experiment. Therefore, we computed rmANOVAs for reported outcomes and memory performance, using string length and experimental half (first or second) as within-subject factors. Significant two-way interactions were followed up by two-tailed paired-samples *t*-tests.

#### Manipulation check

Memory performance significantly decreased with increasing number of letters (two letters: *M* = 0.95, SD = 0.05; five letters: *M* = 0.88, SD = 0.10; eight letters: *M* = 0.65, SD = 0.13), *F*(1.71, 66.66) = 147.31, *p* < 0.001, *η*_p_^2^ = 0.79. Memory performance exceeded chance level for each letter string condition (two letters: *t*(39) = 84.09, *p* < 0.001, *d*_*z*_ = 13.30; five letters: *t*(39) = 35.61, *p* < 0.001, *d*_*z*_ = 5.63; eight letters: *t*(39) = 15.83, *p* < 0.001, *d*_*z*_ = 2.50). Table 1 in the Appendix shows mean medians and standard deviations for recollection time and die roll and report time. Both temporal measures decreased significantly with increasing number of letters (recollection time: *F*(1.64, 64.13) = 72.64, *p* < 0.001, *η*_p_^2^ = 0.65; die roll and report time: *F*(1.47, 57.47) = 54.70, *p* < 0.001, *η*_p_^2^ = 0.58).

**Table 1:** Means (standard deviations in brackets) of medians of recollection and die roll and report time (ms) for letter string lengths in Experiment 1

Letter string length	Recollection time	Die roll and report time
Two letters	1346.85 (342.73)	2001.51 (1133.53)
Five letters	1873.84 (468.16)	2705.19 (1148.40)
Eight letters	2050.30 (536.22)	3140.94 (1013.86)

#### Lying behavior

**Fig. 1:**
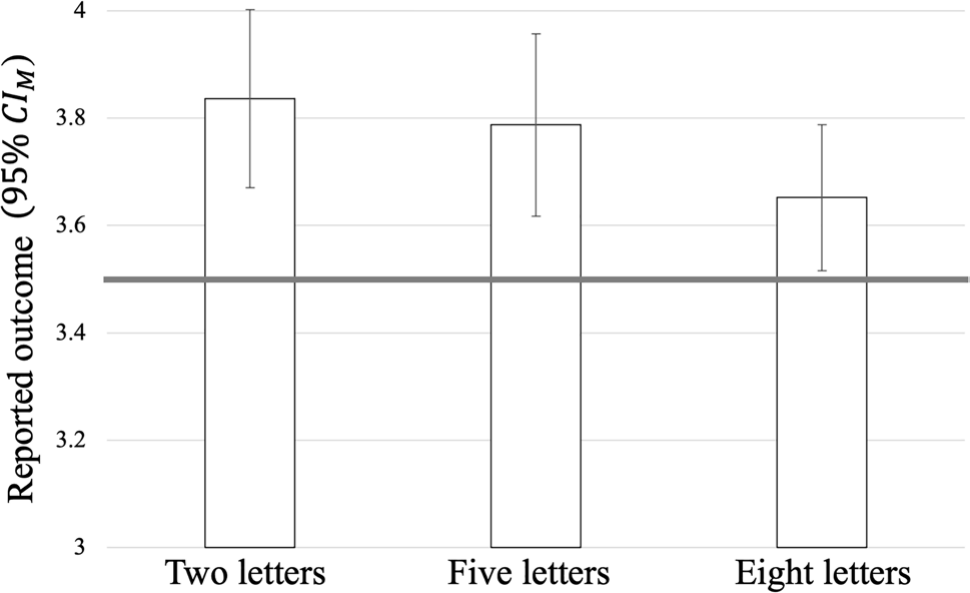
Mean reported outcome for each letter string condition in Experiment 1. The bold horizontal line depicts the chance value of 3.5. Error bars represent the 95% confidence intervals of the individual means (CI_M_)

Reported outcomes significantly decreased when the number of letters increased (see Fig. 1), *F*(1.74, 67.67) = 4.18, *p* = 0.024, *η*_p_^2^ = 0.10, and significantly higher outcomes than 3.5 were reported for each letter string length (two letters: *t*(39) = 4.11, *p* < 0.001, *d*_*z*_ = 0.65; five letters: *t*(39) = 3.42, *p* = 0.002, *d*_*z*_ = 0.54; eight letters: *t*(39) = 2.26, *p* = 0.029, *d*_*z*_ = 0.36).

#### Exploratory temporal development

Memory performance significantly improved in the second half of the experiment as compared to the first half (first half: *M* = 0.80, SD = 0.13, second half: *M* = 0.85, SD = 0.10), *F*(1, 39) = 14.12, *p* = 0.001, *η*_p_^2^ = 0.27. The main effect of string length paralleled the findings of the manipulation checks, *F*(1.71, 66.66) = 147.31, *p* < 0.001, *η*_p_^2^ = 0.79, and no significant interaction effect emerged, *F*(1.69, 65.90) = 1.90, *p* = 0.164, *η*_p_^2^ = 0.05.

Mean reported outcomes showed a non-significant trend towards a decrease from the first to the second half of the experiment (first half: *M* = 3.82, SD = 0.56; second half: *M* = 3.70, SD = 0.59), *F*(1, 39) = 4.02, *p* = 0.052, *η*_p_^2^ = 0.09. The main effect of string length paralleled the main findings, *F*(1.74, 67.67) = 4.18, *p* = 0.024, *η*_p_^2^ = 0.10, whereas the interaction did not approach significance, *F*(2, 78) = 1.05, *p* = 0.355, *η*_p_^2^ = 0.03.

### Discussion

Higher cognitive load indeed decreased dishonesty significantly, corroborating recent findings (Van’t Veer et al., 2014). This effect emerged even though participants reported many die rolls, illustrating the pervasively increased need for cognitive resources when lying (Debey et al., 2014; Foerster et al., 2019). Moreover, we observed dishonesty for all letter string lengths, for each of which memory performance exceeded chance level. In principle, these results support our assumption that dishonesty only occurs if sufficient cognitive capacity is available. However, this outcome does not necessarily imply that dishonesty vanishes if cognitive capacity is maximally taxed. Consequently, in Experiment 2, we further increased cognitive load to answer this question.

An alternative explanation for the positive main effect of cognitive load on lying might be a strategic approach of participants. In the current experimental setup, participants could only earn money for a reported outcome if they remembered the letter string of the same trial correctly. Therefore, the probability of indeed receiving the payoff for the reported outcome was higher in the low load condition. Consequently, participants might have lied more when being confronted with shorter letter strings, as they could be quite sure that they would receive the money. To control for this potential confound, the total payment was based on the overall memory performance instead of each single trial in Experiment 2. Therefore, there was no strategic reason to lie more in one condition than in the others.

Our explorative analyses revealed a descriptive, non-significant tendency for participants to lie less in the second than in the first half of the study. For one, participants might have allocated more cognitive resources to the memory task, reflected by an improvement of memory performance over the course of the experiment. However, practice should also improve memory performance, which should rather leave more cognitive resources available for lying. As such, we think that the descriptive trend in the temporal development of lying behavior more likely roots in adaptation to incentives for dishonesty and we investigated this matter more thoroughly in Experiment 2.

## Study 2

In Experiment 2, we expanded the cognitive load manipulation of the previous experiment and excluded a potential alternative explanation of the previous findings by rendering payoff contingent on overall memory performance instead of the performance in each single trial.

In addition to these technical refinements, we built on the explorative analyses of the first experiment by studying the impact of different incentive structures on the temporal development of dishonesty. This agenda resonates with previous work on the question of how initial minor acts of dishonesty can pave the way for large moral transgressions over time (e.g., Welsh et al., 2015). Focusing specifically on dishonesty, there is evidence that the extent of self-serving lying increases over time which is explained as a habituation to the negative affective response induced by dishonest behavior (Garrett et al., 2016). These results stand in contrast to our exploratory findings in Experiment 1, indicating a descriptive trend towards a decline of dishonesty over time. An explanation for those diverging results might lie in different incentive calculations. Financial reward in Garrett et al. (2016) was only based on one random trial, whereas it was calculated from all correct trials in our first experiment. Therefore, it is plausible that the outcome of our first experiment was the result of an adaptation to the reward over time that counteracted a decline of negative affect. To test this assumption, we introduced both incentive structures in Experiment 2, aiming for different levels of adaptation to the incentive. We hypothesized that reported outcomes would decrease over time for the incentive structure in which all trials counted for the bonus but would stay stable if only one random trial determined financial payoff.

### Method

#### Participants and design

The experiment was preregistered (https://osf.io/63hsy). We conducted a power analysis for which we assumed an effect size of *d*_*z*_ = 0.40 for the impact of cognitive load on lying, which is the average of the effect size in Experiment 1 and in prior research (Van’t Veer et al., 2014). Therefore, we aimed at collecting data of 50 participants for a power of 0.80 in a within-subjects design (*α* = 0.05, two-tailed testing; calculated with the power.t.test function in the statistics package R, version 4.0.3). As determined in advance, we excluded data of participants who showed insights into the experiment’s rationale which was the case for three persons. Moreover, one participant refused consent to further data use after the debriefing. Therefore, our final sample consisted of 46 participants (31 females, 1 diverse; 3 left handers, 1 ambidextrous; age: *M* = 23 years, SD = 8 years). We mostly recruited psychology students of the local university, earning course credit for their participation and an additional financial bonus of five euros.

#### Procedure and materials

The experiment was conducted online. This decision was not part of the preregistration but a consequence of rapidly increasing COVID-19 cases and therefore closed labs at the time of data collection (November to December 2020). In contrast to the first experiment, the letter strings for the secondary task consisted of either 5, 8 or 11 lowercase letters. To provoke different levels of adaptation to reward, we varied the bonus calculation between both experimental halves. In one half, the payoff for each of both blocks was calculated as the average reported outcome in this block multiplied by the share of correctly remembered letters (i.e., “all trials paid”). In the other half, the additional payment for each block only depended on the reported outcome of one random trial, again multiplied by the share of correctly remembered letters (i.e., “one trial paid”). Therefore, both incentive structures allowed the participants to earn a payoff between zero and six euros, however, the final bonus was described as the average of all four blocks. The order of incentive structures was balanced over participants.

The experimental procedure was similar to the first experiment. We advised participants to prepare a regular six-sided die and a cup for the die task. After agreeing to the experimental terms, participants created an individual code for compensation. In addition to indicating age, gender as well as handedness, they answered two questions regarding the color of their die and cup. These questions should prevent participants from not using a die at all. Next, the instructions regarding the exact trial sequence and the two different incentive structures as well as exemplary calculations for these incentive structures were presented. Afterwards, participants carried out six practice trials to get used to the setup.

The procedure of each trial was similar to Experiment 1. For both incentive structures, there were two consecutive blocks of 45 trials, 15 for each letter string condition in random order. After each block, there was a self-paced break and participants were informed about the bonus they had earned in the previous block. Moreover, before each block, the respective incentive structure for the following trials was explained. At the end of the experiment, the final bonus was presented. Participants answered two open-ended questions regarding the assumed purpose of the study and unusual observations. Finally, they were debriefed regarding the real study purpose and each participant received the same bonus payment.

### Results

#### Data treatment and analyses

Raw data, the analysis syntax and programming files are available in the OSF repository of this study (https://osf.io/bp8vt/). We did not analyze practice trials. Due to a technical error, negative response times were collected for a few trials, and we excluded these trials from all secondary analyses of response times.

Statistical analyses were similar as in Experiment 1, except for the following changes. We employed rmANOVAs with the factors incentive structure (all trials paid vs. one trial paid), block within incentive structure (first vs. second) and letter string length (5 vs. 8 vs. 11 letters) for the same dependent measures as in Experiment 1. Any significant three-way interaction was further explored in separate two-way rmANOVAs for both incentive structures. If those analyses revealed a significant two-way interaction, we calculated additional two-tailed paired-samples *t*-tests comparing the dependent variable between both blocks for each string length.

To investigate the temporal development within blocks, we compared reported outcomes and memory performance between both halves of each block and incentive structure. Therefore, we computed a rmANOVA for reported outcomes and memory performance, using incentive structure, block within incentive structure and block half within incentive structure (first vs. second) as factors.^2^

#### Manipulation check

Memory performance significantly decreased with longer letter strings (five letters: *M* = 0.86, SD = 0.13; eight letters: *M* = 0.65, SD = 0.13; eleven letters: *M* = 0.54, SD = 0.13), *F*(1.75, 78.83) = 189.16, *p* < 0.001, *η*_p_^2^ = 0.81, and was significantly higher than guessing rate in all letter string conditions (five letters: *t*(45) = 26.84, *p* < 0.001, *d*_*z*_ = 3.96; eight letters: *t*(45) = 15.96, *p* < 0.001, *d*_*z*_ = 2.35; eleven letters: *t*(45) = 11.42, *p* < 0.001, *d*_*z*_ = 1.68). Neither the main effect of incentive structure (all trials paid: *M* = 0.69, SD = 0.11; one trial paid: *M* = 0.68, SD = 0.13), *F*(1, 45) = 1.84, *p* = 0.182, *η*_p_^2^ = 0.04, nor the main effect of block within incentive structure (block 1: *M* = 0.68, SD = 0.11; block 2: *M* = 0.69, SD = 0.13), *F* < 1, nor any interactions were significant, *F*s < 1.

Tables 2 and 3 in the Appendix show means and standard deviations of each experimental cell regarding recollection time as well as die roll and report time. For longer letter strings, we found a significant increase of recollection time, *F*(1.29, 58.07) = 6.82, *p* = 0.007, *η*_p_^2^ = 0.13, as well as of die roll and report time, *F*(1.30, 58.31) = 46.00, *p* < 0.001, *η*_p_^2^ = 0.51. Moreover, both measures significantly decreased from the first to the second block within both incentive structures (recollection time: *F*(1, 45) = 4.53, *p* = 0.039, *η*_p_^2^ = 0.09; die roll and report time: *F*(1, 45) = 36.58, *p* < 0.001, *η*_p_^2^ = 0.45) but did not differ significantly between incentive structures (recollection time: *F*(1, 45) = 2.25, *p* = 0.141, *η*_p_^2^ = 0.05; die roll and report time: *F* < 1). The two-way interactions were not significant in either measure, *F*s ≤ 2.29, *p*s ≥ 0.137, *η*_p_^2^ ≤ 0.05. The three-way interaction of all factors did not reach significance for die roll and report time, *F* < 1, but for recollection time, *F*(2, 90) = 3.35, *p* = 0.039, *η*_p_^2^ = 0.07. For the all trials paid condition, the main effect of string length was significant, *F*(1.50, 67.31) = 4.39, *p* = 0.025, *η*_p_^2^ = 0.09, but not the main effect of block or the interaction of letter string condition and block, *F*s < 1. Regarding the one trial paid condition, we found two significant main effects (letter string length: *F*(1.42, 63.95) = 6.51, *p* = 0.007, *η*_p_^2^ = 0.13; block: *F*(1, 45) = 6.26, *p* = 0.016, *η*_p_^2^ = 0.12) as well as a significant interaction of both factors, *F*(2, 90) = 4.20, *p* = 0.018, *η*_p_^2^ = 0.09. We only observed a significant difference between the first and second block for the eight letters condition, *t*(45) = 3.22, *p* = 0.002, *d*_*z*_ = 0.47, but not for five, |*t|*< *1*, or eleven letters, *t*(45) = 1.33, *p* = 0.189, *d*_*z*_ = 0.20.

**Table 2:** Means (standard deviations in brackets) of medians of recollection time (ms) for letter string lengths, incentive structures and blocks in Experiment 2

Letter string length	All trials paid		One trial paid	
	Block 1	Block 2	Block 1	Block 2
Five letters	1824.95 (542.69)	1737.94 (584.70)	1827.24 (506.23)	1801.80 (549.46)
Eight letters	1981.86 (685.99)	2005.26 (832.79)	2252.08 (808.01)	1983.91 (809.78)
Eleven letters	2024.35 (958.37)	1994.51 (1,007.14)	2148.50 (903.84)	2038.37 (1,193.21)

**Table 3:** Means (standard deviations in brackets) of medians of die roll and report time (ms) for letter string lengths, incentive structures and blocks in Experiment 2

Letter string length	All trials paid		One trial paid	
	Block 1	Block 2	Block 1	Block 2
Five letters	2109.17 (671.99)	1833.95 (705.11)	2192.30 (823.91)	1953.95 (808.96)
Eight letters	2478.80 (826.91)	2247.60 (807.50)	2594.92 (1060.46)	2294.06 (1012.12)
Eleven letters	2505.91 (902.00)	2384.41 (867.43)	2630.71 (1143.17)	2420.72 (975.09)

#### Lying behavior

**Fig. 2:**
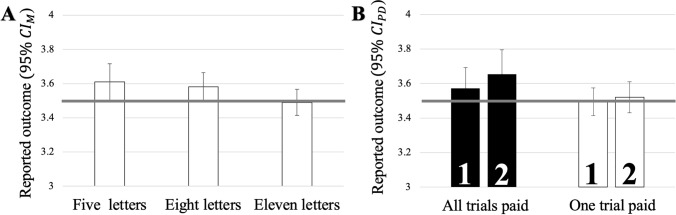
**A** Mean reported outcome for each string length in Experiment 2. The bold horizontal line depicts the chance value of 3.5. Error bars represent confidence intervals of the individual means (CI_M_). **B** Mean reported outcome for each combination of incentive structure and block in Experiment 2. The bold horizontal line depicts the chance value of 3.5 and the bold numbers indicate the block within incentive structure. Error bars represent the confidence intervals for paired differences, calculated separately for each incentive structure (CI_PD_; Pfister & Janczyk, 2013)

Figure 2 shows the main results of Experiment 2, whereas Fig. 4 in the Appendix depicts reported outcomes for the full design in detail. Reported outcomes significantly declined if the number of letters increased (see Fig. 2A), *F*(1.67, 75.19) = 3.87, *p* = 0.032, *η*_p_^2^ = 0.08, and only significantly exceeded the expected value for the five letters condition (five letters: *t*(45) = 2.06, *p* = 0.045, *d*_*z*_ = 0.30; eight letters: *t*(45) = 1.99, *p* = 0.052, *d*_*z*_ = 0.29; eleven letters: |*t|*< 1). Neither the main effect of incentive structure, *F*(1, 45) = 3.99, *p* = 0.052, *η*_p_^2^ = 0.08, nor block, *F*(1, 45) = 2.23, *p* = 0.142, *η*_p_^2^ = 0.05, was significant. None of the interactions approached significance either, *F*s ≤ 2.54, *p*s ≥ 0.777, *η*_p_^2^ ≤ 0.01.

#### Exploratory analyses

Table 5 in the Appendix shows reported outcomes and Table 6 in the Appendix shows memory performance for both incentive structures, blocks within incentive structure and block halves. For reported outcomes, neither the main effect of block half, *F* < 1, nor block, *F*(1, 45) = 2.19, *p* = 0.146, *η*_p_^2^ = 0.05, nor incentive, *F*(1, 45) = 4.04, *p* = 0.051, *η*_p_^2^ = 0.08, was significant. None of the interactions was significant, *F*s ≤ 1. Also for memory performance, no main effect (block half and block: *Fs* < 1; incentive: *F*(1, 45) = 1.98, *p* = 0.167, *η*_p_^2^ = 0.04) and no interaction was significant, *F*s ≤ 1.95, *p*s ≥ 0.169, *η*_p_^2^ ≤ 0.04.

### Discussion

In our second experiment, high cognitive load again significantly reduced dishonesty, even though there was no strategic advantage in lying less in the high cognitive load condition. Moreover, for all cognitive load conditions memory performance was significantly higher than guessing rate, whereas we only observed a result pattern in line with dishonesty in the low cognitive load condition. This result indicates that lying may sometimes vanish completely even before a second task fully draws on all available cognitive resources. Moreover, it appears possible that participants’ cognitive resources were indeed completely engaged by the memory task, but that they still remembered letters better than chance by just focusing on a part of the letter string as we only asked for one letter and not the whole string. Furthermore, the baseline extent of dishonesty might differ within and between subjects due to several other factors like personality traits (Pfattheicher et al., 2019) or norm perception (Mitra & Shahriar, 2020). Due to these issues, we conclude that focusing on a specific value of cognitive load above which lying is no longer recognizable is probably not the most promising research approach. Instead, the general effect of cognitive load on dishonesty should be in focus. Consequently, Experiment 3 did not further analyze this issue and only used the shortest and the longest letter strings to maximize the impact of the cognitive load manipulation.

Furthermore, we introduced two incentive structures in which the financial payoff was either based on all trials or only one random trial. We proposed that the first mentioned incentive structure should facilitate reward adaptation and therefore a decline of dishonesty over time relative to the one trial paid condition should be likely. In contrast to this assumption, dishonesty only descriptively increased from the first to the second block within both incentive conditions. Based on evidence of less commitment to the experimental task in online studies of lying behavior (Dickinson & McEvoy, 2021), it is possible that participants simply did not carefully read instructions which is why this manipulation might not have worked as intended. However, this concern seems unlikely against the interactive impact of incentives on recollection time and the descriptive trend towards a main effect on reported outcomes.

For one, we did not replicate a decline of lies over time in the all trials paid condition as we had observed in Experiment 1. Comparing both setups, it has to be noted that we did not present the current score after each trial in Experiment 2 as in Experiment 1, to introduce comparable procedures for both incentive structures here. This monetary reminder, however, may have reinforced the adaptation to rewards significantly in the first experiment. Second, we did not observe an increase in lying over time in the one trial paid condition (Garrett et al., 2016). Due to several differences between the setup of Garrett et al. (2016) and the present experiment (i.e., operationalization of dishonesty, number of trials, two different incentive structures and an additional cognitive load manipulation) it is hard to derive convincing explanations for these inconsistent results. Further exploratory analyses showed that dishonesty within each block declined descriptively for all combinations of block and incentive structure except of a tiny descriptive increase in the first block of the one trial paid condition. Even though this was only a descriptive, non-significant trend, this finding is remarkable as the development between blocks points in the opposite direction. As we did not present the current score after each trial, but only at the end of each block, it is possible that this monetary reminder boosted dishonesty in the short term at the beginning of each block, whereas the motivation to lie declined within each block. This assumption is supported by an increase of unethical behavior due to monetary primes elsewhere (Kouchaki et al., 2013). Experiment 3 will further investigate this issue in an exploratory manner but will only present the current bonus at the end of each incentive structure instead of each block.

## Study 3

Experiment 3 further investigated the influence of cognitive load and varying incentive structures on dishonesty by introducing a condition in which participants could lie to limit a loss instead of maximizing a gain. The concept of loss aversion, which is an integral part of *prospect theory* (Kahneman & Tversky, 1979), proposes that “losses loom larger than gains” (Kahneman & Tversky, 1979, p. 279). This phrase describes the observation that losses are valued as more negative than the equal amount of profit is perceived as positive. Importantly, this effect also arises by only framing a decision in a way that emphasizes either possible losses or gains (Tversky & Kahneman, 1981; for a comprehensive replication see Druckman, 2001). Due to the substantial influence of framing on decision-making, it stands to reason that this effect also needs to be investigated thoroughly in the field of unethical behavior. Human agents regularly face situations in which the motivation for a lie might be to prevent losses instead of receiving gains (e.g., tax fraud or false statements about age for reduced entrance fees). Accordingly, investigating the effect of cognitive load on dishonesty in such situations bears important practical implications and can provide critical information about the generalizability of the findings from Experiment 1 and Experiment 2.

Research of loss aversion indicates that preventing losses is a stronger motivation for a specific behavior than collecting gains (e.g., Gächter et al., 2010). Also in previous studies of dishonesty research, avoiding losses was a greater motivation to lie than the possibility to increase gains (Cameron et al., 2010; Grolleau et al., 2016), specifically in the *die under the cup paradigm* (Schindler & Pfattheicher, 2017). Based on these results, we expected participants to value potential losses as more negative than the same amount of potential gains as positive. Accordingly, we hypothesized that more dishonesty should occur to limit losses than to increase financial gains. Participants collected a financial bonus in the first half of the experiment which they could lose in the second half. However, they had the possibility to restrict losses by lying. Due to a tendency to value objects more if you invested effort to create or obtain them (Norton et al., 2012), this manipulation should further increase loss aversion and consequently dishonesty.

Finally, the combination of cognitive load and loss aversion in one experiment offered the opportunity to investigate the interaction of both factors. Prior research showed that people are only more willing to engage in morally questionable behavior in order to prevent losses if they have to make a decision under time pressure (Kern & Chugh, 2009). Two other studies could not find a significant interaction of cognitive load and loss aversion (Bogliacino & Montealegre, 2020; Guillemette et al., 2014). Consequently, this research question has only been investigated insufficiently and the existing evidence is far from allowing coherent conclusions. However, based on findings that for one, cognitive load increases risk aversion (e.g., Benjamin et al., 2013; for a review, see Deck & Jahedi, 2015) and for another, loss aversion is seen as one of the key influencing factors of risk aversion in theoretical models (e.g., Köbberling & Wakker, 2005), we expected more loss aversion under high cognitive load. Therefore, lying behavior should differ significantly more between the gain and the loss condition if less than more cognitive resources are available.

### Method

#### Participants and design

This experiment was preregistered (https://osf.io/r3c9g). Based on the results of Experiment 2, we expected an effect size of *d*_*z*_ = 0.35 (five vs. eleven letters) in reported outcomes for the cognitive load manipulation. Regarding the impact of the two incentive frames, previous studies found even larger effects (e.g., *d* = 0.56 for Schindler & Pfattheicher, 2017). A power analysis resulted in a sample size of 66 participants for a probability of ≥ 0.80 to detect a significant effect of the cognitive load as well as incentive manipulation in a within-subjects design (*α* = 0.05, two-tailed testing; calculated with the power.t.test function in the statistic package R, version 4.0.3). No data exclusions were necessary, because no participant guessed our hypotheses or refused consent to data use after debriefing. The sample (48 females; 3 left handers, 1 ambidextrous; age: *M* = 27 years, SD = 9 years) was recruited from the local university’s participant pool for online participation and every participant earned five euros for participation plus an additional bonus of two euros.

#### Procedure and materials

Experiment 3 was also conducted online because of the ongoing pandemic. Again, participants were asked to prepare a regular six-sided die and a cup for the die task. In contrast to Experiment 2, the stimuli for manipulating cognitive load only consisted of five or eleven letters but we now compared a gain and a loss framing. Participants were told that they could earn an additional bonus. For the first half of the experiment, this bonus was allegedly calculated as the reported outcome in one random trial multiplied by the share of correctly remembered letters (i.e., “gain incentive”). In the second half, participants were informed that the bonus now equalled the reported outcome in one random trial multiplied by the share of incorrectly remembered letters and that the result of this calculation would be subtracted from their collected bonus in the first half (i.e., “loss incentive”). Participants were informed that losses were capped at the level of the previous gain so that the final bonus could supposedly be in the range of zero and six euros. Again, we implemented exemplary calculations of both incentive structures before the respective experimental blocks. In addition, participants had to calculate two exemplary bonuses correctly to start the experiment and proceed after the first half. There was no time limit for doing so and in case of a wrong response they could try again as often as they liked.

Each block now consisted of 30 randomized trials, again 15 for each of the two letter string conditions. In comparison to the second experiment, participants were only informed about the earned bonus at the end of each incentive structure instead of at the end of each block to not distort the temporal development of lying behavior within each incentive structure. Moreover, we did not show participants their real bonus after the first half, but everyone was told that they earned 4.37€. We specifically selected a comparably high but still realistic value because the maximum loss for the second half was restricted to this this value and a very low amount of potential loss might prevent dishonesty.

At the end of the experiment the final bonus was presented. Again, the displayed amount of two euros was identical for all participants. They further answered three open-ended questions. In addition to the questions regarding the purpose of the study and unusual observations, we asked for any strategy use. Finally, participants were debriefed about the real study subject as well as the faked bonus calculations.

### Results

#### Data treatment and analyses

Raw data, the analysis syntax and programming files are available in the OSF repository (https://osf.io/c9726/). We did not analyze practice trials. We analyzed the same dependent variables as in the former experiment in separate two-way rmANOVAs with the factors incentive structure (gain vs. loss) and letter string length (five vs. eleven letters). Due to the two different incentive structures, dishonesty was indicated by an upward deviation of reported outcomes from the expected value (3.5) in the gain condition but a downward deviation from that value in the loss condition. To facilitate statistical analyses, we transformed the dependent variable so that positive values indicated lying for both conditions. Therefore, we subtracted the expected value from average reported outcomes of each participant in the gain condition and the mean reported outcomes from the expected value in the loss condition. Significant interactions of letter string length and incentive structure were further analyzed by two-tailed paired-samples *t*-tests. We further tested the transformed reported outcome against the expected value (0) for each design cell. Finally, we calculated an exploratory two-tailed *t*-test for each incentive structure comparing transformed reported outcomes of both letter string conditions.

In a secondary analysis, we examined the temporal development of lying behavior in a three-way rmANOVA on transformed reported outcomes using the experimental block within incentive structure (first vs. second) as an additional within-subject factor next to letter string condition and incentive structure. Moreover, the same analysis was calculated for memory performance. We followed up on significant interactions as in the preceding experiments.

#### Manipulation check

Memory performance significantly decreased for longer letter strings (five letters: *M* = 0.86, SD = 0.13; eleven letters: *M* = 0.58, SD = 0.16), *F*(1, 65) = 280.18, *p* < 0.001, *η*_p_^2^ = 0.81, and significantly improved in the loss condition (gain: *M* = 0.71, SD = 0.14; loss: *M* = 0.73, SD = 0.14), *F*(1, 65) = 4.27, *p* = 0.043, *η*_p_^2^ = 0.06, whereas the interaction was not significant, *F* < 1.

Table 4 in the Appendix shows means and standard deviations for die roll and report time as well as recollection time. Both measures significantly increased for long as compared to short letter strings (die roll and report time: *F*(1, 65) = 52.91, *p* < 0.001, *η*_p_^2^ = 0.45; recollection time: *F*(1, 65) = 15.03, *p* < 0.001, *η*_p_^2^ = 0.19), and were reduced in the loss framing relative to the gain framing (die roll and report time: *F*(1, 65) = 36.50, *p* < 0.001, *η*_p_^2^ = 0.36; recollection time: *F*(1, 65) = 52.07, *p* < 0.001, *η*_p_^2^ = 0.46). There was no significant interaction in either analysis (die roll and report time: *F*(1, 65) = 1.53, *p* = 0.220, *η*_p_^2^ = 0.02; recollection time: *F* < 1).

**Table 4:** Means (standard deviations in brackets) of medians of die roll and report time as well as recollection time (ms) for letter string lengths and incentive structures in Experiment 3

Letter string length	Die roll and report time		Recollection time	
	Gain	Loss	Gain	Loss
Five letters	2359.98 (1107.30)	2024.52 (1019.99)	2225.98 (892.47)	1877.61 (755.60)
Eleven letters	2736.71 (1157.94)	2334.92 (1111.60)	2570.58 (1437.67)	2242.60 (1155.84)

#### Lying behavior

**Fig. 3:**
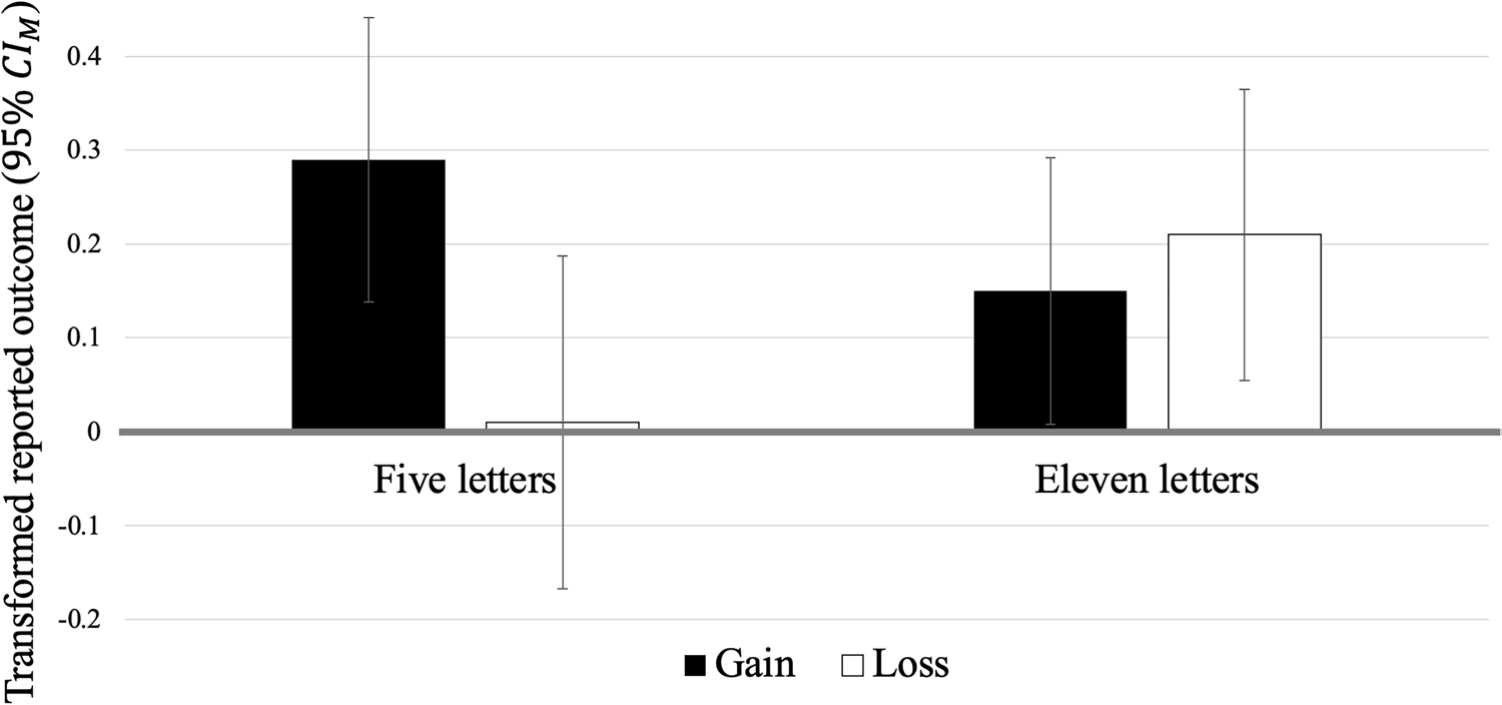
Mean transformed reported outcome for each letter string condition and incentive structure in Experiment 3. The bold horizontal line depicts the chance value of 0. Error bars represent the 95% confidence intervals of the individual means (CI_M_)

**Fig. 4:**
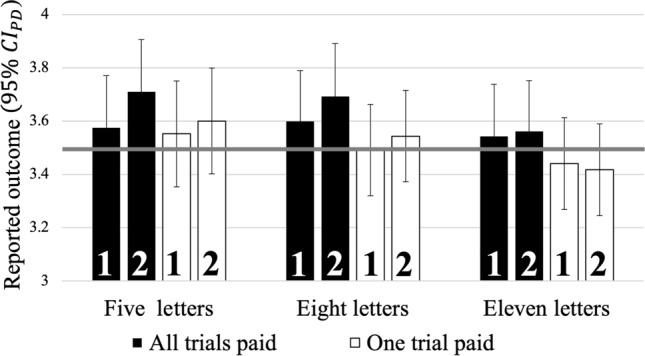
Mean reported outcome for each combination of letter string length, incentive structure and block in Experiment 2. The bold horizontal line depicts the expected value of 3.5 and the bold numbers represent the block within incentive structure. Error bars represent the confidence intervals of the paired differences calculated separately for each combination of letter string length and incentive structure (CI_PD_)

Figure 3 shows the main results of the experiment. The rmANOVA yielded a significant intercept, *F*(1, 65) = 10.10, *p* = 0.002, *η*_p_^2^ = 0.14, indicating overall lying because transformed reported outcomes were larger than zero. Furthermore, transformed reported outcomes were not significantly affected by the number of letters (five letters: *M* = 0.15, SD = 0.67; eleven letters: *M* = 0.18, SD = 0.60), *F* < 1, or by incentive structure (gain: *M* = 0.22, SD = 0.60; loss: *M* = 0.11, SD = 0.68), *F*(1, 65) = 1.72, *p* = 0.195, *η*_p_^2^ = 0.03. The interaction of letter string length and incentive structure was significant, however, *F*(1, 65) = 6.28, *p* = 0.015, *η*_p_^2^ = 0.09. As depicted in Fig. 3, transformed reported outcomes were higher in the gain than in the loss condition for short letter strings, *t*(65) = 2.39, *p* = 0.020, *d*_*z*_ = 0.29, whereas there was no significant difference for long letter strings, |*t*|< 1. Moreover, regarding both incentive structures, transformed reported outcomes were significantly above zero for long letter strings (gain: *t*(65) = 2.08, *p* = 0.042, *d*_*z*_ = 0.26; loss: *t*(65) = 2.71, *p* = 0.009, *d*_*z*_ = 0.33) as well as for short letter strings in the gain condition, *t*(65) = 3.87, *p* < 0.001, *d*_*z*_ = 0.48, but not in the loss condition, |*t|*< 1.

#### Exploratory analyses

In the gain condition, transformed reported outcomes were significantly higher for short than for long letter strings (see Fig. 3), *t*(65) = 2.25, *p* = 0.028, *d*_*z*_ = 0.28, whereas the opposite pattern could be observed for the loss condition, *t*(65) = − 2.09, *p* = 0.041, *d*_*z*_ = − 0.26.

**Fig. 5:**
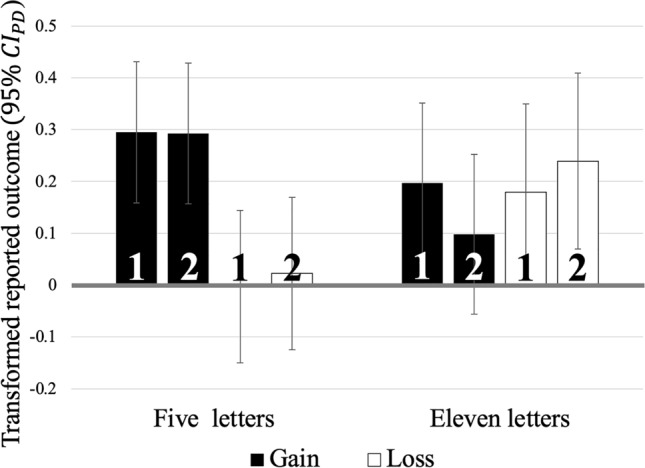
Mean reported outcome for each combination of letter string length, incentive structure and block in Experiment 3. The bold horizontal line depicts the expected value of 0 and the bold numbers represent the block within incentive structure. Error bars represent the confidence intervals of the paired differences calculated separately for each combination of letter string condition and incentive structure (CI_PD_)

Figure 5 in the Appendix depicts transformed reported outcomes for the different experimental cells including block within incentive structure. None of the main effects was significant, *F*s ≤ 1.72, *p*s ≥ 0.195, *η*_p_^2^ ≤ 0.03, and only letter string length and incentive structure interacted significantly as in the previous main analysis, *F*(1, 65) = 6.28, *p* = 0.015, *η*_p_^2^ = 0.09. Transformed reported outcomes were higher in the gain than in the loss condition for short letter strings, *t*(65) = 2.39, *p* = 0.020, *d*_*z*_ = 0.29. For longer letter strings, no significant difference was observed, |*t|*< 1. The remaining interactions were not significant, *F*s ≤ 1.13, *p*s ≥ 0.292, *η*_p_^2^ ≤ 0.02.

Table 7 in the Appendix illustrates memory performance for the different experimental cells including block within incentive structure. The main effects of letter string length, *F*(1, 65) = 280.18, *p* < 0.001, *η*_p_^2^ = 0.81, and incentive structure, *F*(1, 65) = 4.27, *p* = 0.043, *η*_p_^2^ = 0.06, were significant, but not the main effect of block, *F*(1, 65) = 1.97, *p* = 0.165, *η*_p_^2^ = 0.03. Neither two-way nor three-way interactions were significant, *F*s ≤ 2.04, *p*s ≥ 0.158, *η*_p_^2^ ≤ 0.03.

### Discussion

Experiment 3 built on the results of Experiments 1 and 2 and further investigated the effect of cognitive load on dishonesty. By introducing a condition in which participants could lie to limit a loss instead of increasing a financial gain, the effect of different incentive frames was analyzed. Discordant to our assumption and the outcome of Experiments 1 and 2 as well of other researchers (Van’t Veer et al., 2014), higher cognitive load did not reduce dishonesty. Only exploratory analyses showed the proposed effect for the gain condition, but the opposite pattern if participants faced losses.

Also contrary to our assumptions, facing losses instead of gains did not increase lying behavior as suggested by preceding studies in the field of ethical behavior (Cameron et al., 2008; Grolleau et al., 2016; Schindler & Pfattheicher, 2017) and general findings about incentive framing (Gächter et al., 2010; Kahneman & Tversky, 1979). This is especially surprising as our setup should have had the potential to further increase loss aversion because participants could lose money for which they invested effort before (Norton et al., 2012). Instead, gains led to more lies than losses, at least under low cognitive load. This surprising interplay of cognitive load and incentive structure makes it complicated to evaluate our general hypothesis whether the framing effect would be larger under high than low cognitive load. On the one hand, we observed a reversed framing effect that was larger under low than high cognitive load. On the other hand, we specified that facing losses should increase dishonesty especially under high cognitive load due to a higher risk aversion (Benjamin et al., 2013; Köbberling & Wakker, 2005). Post hoc analyses showed significant more lying behavior for the loss condition under high compared to low cognitive load, which might indeed be a consequence of increased loss aversion due to scarce cognitive capacity.

Moreover, the non-significant effect of the loss frame on lying behavior could be due to some characteristics of our setup. It is important to mention that the bonus did not only depend on the reported outcomes and therefore honesty but also on memory performance. As the share of correctly remembered letters was significantly higher in the loss condition, it is plausible that participants indeed behaved loss-aversely. As there were two options to limit losses, however, they decided for the more ethical one—to put more effort into the memory task—to keep a positive self-view (Mazar et al., 2008). This assumption is supported by the finding that people show more effort to reduce losses than to increase gains (Farinha & Maia, 2021). However, as the order of incentive structures was identical for all participants, the improvement in the loss condition could also be a consequence of practice effects over time, as observed in Experiment 1.

In addition, the stakes in our setup were quite small. Findings of reversed loss aversion for small incentives (Harinck et al., 2007) could partially explain our results, even though other studies provided evidence in favor of increased dishonesty because of loss aversion also for small stakes (e.g., Cameron et al., 2010; Schindler & Pfattheicher, 2017). The exact way the bonus was calculated offers further clues for an explanation of the unexpected results. Participants could allegedly earn up to six euros in the gain condition, but they could only lose the win of the first half in the loss condition. As we set the bonus of the first half to a fixed value of 4.37€ for all participants, the maximum possible loss was below the highest possible gain which could have reduced loss aversion. Furthermore, the win was allegedly calculated as the reported outcome in one random trial multiplied by the share of correctly remembered letters, whereas the possible loss allegedly equalled the product of one random reported outcome and the share of incorrectly remembered letters. Only if exactly half of the letter strings had been recollected correctly, the result of this calculation would be, assuming the same amount of dishonesty, equal for both incentive structures. However, as memory performance across all conditions was much higher than 0.50 (*M* = 0.72), equal lying behavior and memory performance in both conditions would not have resulted in equal outcomes. If participants noticed these asymmetries, they might have evaluated high losses less likely than high gains. Moreover, such an effect might have been additionally magnified by the improved memory performance in the loss condition.

Even though the experiment does not deliver a clear picture on the exact interplay of loss and gain frames with cognitive load in lying, its results demonstrate that these two aspects jointly promote or diminish lying and points to several further intriguing research questions. For example, it seems worthwhile to explore if agents are more willing to invest more cognitive effort in a task to prevent losses than to lie for the same goal. To investigate the interplay of incentive framing and cognitive load in lying more precisely, it might be helpful to employ a more stringently controlled experimental design to eliminate the influence of confounding variables even further.

## General discussion

We investigated the cognitive and motivational foundations of self-serving dishonesty in three experiments. Lying behavior was operationalised by the *die under the cup paradigm* (Fischbacher & Föllmi-Heusi, 2013). Participants reported the outcome of private die rolls with specific outcomes connected to a higher financial payoff. Accordingly, misreporting to increase the payoff was possible while there was no risk of detection.

Crucially, we applied a dual-task paradigm in which participants had to remember letter strings while conducting the die task (Van’t Veer et al., 2014). Manipulating the length of the letter strings allowed us to induce conditions of variable cognitive load and thus interfere with effortful processing in conditions of high load (Experiment 1: two, five or eight letters; Experiment 2: five, eight or eleven letters; Experiment 3: five or eleven letters). Moreover, Experiment 2 and 3 used two different incentive structures. In Experiment 2, we varied whether the final payoff was determined by all trials or only one random trial to manipulate a possible adaptation to reward over time. Experiment 3 framed the incentive for lying as either a gain or a loss.

For each experiment, longer letter strings significantly reduced the share of correctly remembered letters and increased the required time for the die task and recollecting the letter string. Based on the increase in temporal measures and above-chance performance in all conditions, it can be assumed that lower memory performance was not only a result of participants pressing random keys but higher cognitive load throughout the task. Assuming dishonesty as cognitively demanding, we proposed less dishonesty under high cognitive load for each study. This assumption was supported by the results of Experiments 1 and 2 but could not be validated in Experiment 3. However, post hoc analyses showed that honesty was indeed promoted by cognitive load in the gain condition of Experiment 3, whereas a significant effect in the opposite direction could be found for the loss condition. The finding of less available cognitive resources limiting dishonesty is in accordance with prior research using a similar setup (Van’t Veer et al., 2014). That high cognitive load increased dishonesty in the loss condition in Experiment 3 of course contradicts those outcomes at first glance. Future research should examine whether this surprising result can be traced back to an increase of loss aversion under high cognitive load due to more risk aversion (Benjamin et al., 2013; Köbberling & Wakker, 2005). Therefore, the general impact of cognitive capacity on loss aversion should also be put to closer investigation as the current state of research is quite inconsistent (Bogliacino & Montealegre, 2020; Guillemette et al., 2014; Kern & Chugh, 2009). Moreover, the inconsistency regarding the effect of cognitive load on dishonesty across our experiments could partly reflect limitations in statistical power. Based on pilot data (Experiment 1) and previously reported effect sizes (Van’t Veer et al., 2014), we had assumed a small effect of *d*_*z*_ = 0.40 for our power calculations when planning Experiment 2 and a similarly small effect of *d*_*z*_ = 0.35 when planning Experiment 3, resulting in comparable sample sizes across all three studies. The small effect size might partly derive from the noise inherent in the outcome measure of die rolls, which also comes with high variance even for honest die roll reports. If this estimate is correct, however, then one might expect to observe negative results for individual studies when running a series of three studies with an effective power of roughly 80% each. Whether the deviating outcome in the loss condition of Experiment 3 was due to a Type II error or whether it reflects an actual peculiarity of lying under loss aversion, therefore, remains to be tested in future work.

Next to the automaticity of dishonesty and the influence of different incentive structures, we analyzed the temporal development of lying behavior across each experiment. In none of our studies, we found a significant change of dishonesty during the experiment. This finding does not support the assumption of an escalation of self-serving dishonesty over time (Garrett et al., 2016; Ting, 2018), at least not on the timescale investigated here. Neither manipulations of reward structures that aimed at changing adaptation to reward (Experiment 2), nor presenting the bonus at the end of each incentive structure (Experiment 3) instead of each block (Experiment 2) did affect this development particularly. Importantly, none of our experiments focused solely on the temporal development of dishonesty but we also employed varying levels of cognitive load in each study. It is therefore possible that our load manipulation added proactive interference across the experiments (e.g., Shipstead & Engle, 2013), which might have reduced dishonesty over time counteracting any potential escalation effects. As the evidence regarding this research question is scarce, further investigations seem urgently needed. A promising perspective could be offered by expanding measuring methods, for example to assess affective responses. This would be important to examine whether the negative affective response induced by dishonesty (Gamer et al., 2006) indeed declines over time. If this is not the case, the theoretical basis for an increase of self-serving dishonesty over time would lose ground. A comparably simple approach could be the combination of the classic *die under the cup paradigm* (Fischbacher & Föllmi-Heusi, 2013) and the measurement of skin conductance responses to approach this question more thoroughly.

To gain deeper insights into the effect of cognitive load on dishonesty, we conducted exploratory analyses of the distribution of lying behavior within our samples (see Appendix 4 for a detailed report). Prior research indicates that the vast majority of lies are told by only a few liars (Gneezy et al., 2018; Serota & Levine, 2015; Serota et al., 2010). Surprisingly, our analyses did not only point to a considerable number of such prolific liars but they also revealed outliers in the opposite direction, i.e., a few participants who reported outcomes significantly below the expected value. This finding could be problematic for our conclusion that cognitive load promoted honesty. Alternatively, reduced mental capacity might increase the use of strategic approaches. For instance, participants might have been more tempted to not roll the die at all and instead they might have been trying to make up some kind of random sequence, accidentally reporting low outcomes relatively frequently. However, neither the share of low outliers nor the share of high outliers varied with cognitive load across experiments (see Appendix 4). Further, the exclusion of the strongest low outliers from our analyses did not change any statistical decision on the impact of cognitive load on die reports. By additionally excluding participants who showed pronounced lying behavior, we explored whether cognitive load changes lying behavior for infrequent liars. After corresponding data exclusions, none of our experiments showed a significant effect of cognitive load on dishonesty. A detailed look at the effect size with and without this outlier exclusion reveals that the influence of cognitive load on dishonesty was indeed smaller on a descriptive level for the dataset without outliers in Experiment 1, whereas in the other two experiments effects remained similar suggesting that a drop in power might be responsible for the absence of effects. We therefore propose that our finding of cognitive load promoting honesty is not necessarily limited to prolific liars but might be strongly driven by this group. This is an intriguing finding considering that these prolific liars might have set their mind to frequent lying. Still, they seem to suffer from the cognitive costs of generating dishonest responses, rather than providing prepared lies easily. Within-subject designs enable the observation of dishonest behavior on multiple occasions for each participant, allowing to take such interpersonal variation into account.^3^ Future research of dishonesty should leverage this advantage to pinpoint all facets of the cognitive underpinnings of dishonesty.

Our insights are not only intended to inform the research community but also bear practical implications for the field of lie detection. If cognitive load directly affects dishonesty, limiting available cognitive resources should be a suitable approach to detect dishonesty in real-world scenarios like police interrogations (Vrij et al., 2017; Walczyk et al., 2013). High cognitive load should either directly reduce dishonesty, or it should make it more cognitively challenging to produce a lie, which in turn might increase the chance to expose a liar. However, also for lie detection, the detailed interplay of incentive frame and cognitive load can be critical. For instance, the motivation to lie for a criminal suspect is probably to prevent a fine (loss of money) or imprisonment (loss of freedom). If cognitive load indeed increases dishonesty when facing potential losses, the benefit of imposing cognitive load for lie detection in such scenarios would be highly questionable.

Taken together, most of our results as well as prior research (Van’t Veer et al., 2014) indicate that lying is cognitively demanding and honesty the automatic response. Moreover, as we did not instruct participants to lie but investigated motivated, self-serving dishonesty, our results extend previous findings on performance measures in forced-choice settings to a situation that likely generalizes beyond the lab. However, the finding of more lies under high cognitive load in the loss condition of Experiment 3 requires further investigation. In general, including further factors like temporal development or varying incentive structures in the experimental setup seems to decrease the consistency of the results notably. This inconsistency also applies to other studies in this field, for example research investigating the automaticity of lying behavior by manipulating time pressure (Van der Cruyssen et al., 2020). Moreover, the effect of loss framing on dishonesty does not seem to be as simple as earlier research indicates or could also be limited to one-shot setups (e.g., Schindler & Pfattheicher, 2017). Consequently, more research with varying study designs and stake sizes is necessary to make clear statements about the cognitive and motivational underpinnings of lies. Moreover, technological innovations that allow to detect dishonesty for specific trials, for example through customized dies (as used in Kröll & Rustagi, 2016) or hidden cameras (Du et al., 2021) are auspicious possibilities for future research. That way, the advantages of instructed and motivational designs could be combined which would result in high internal as well as external validity at the same time.

## Figures and Tables

**Fig. 6:**
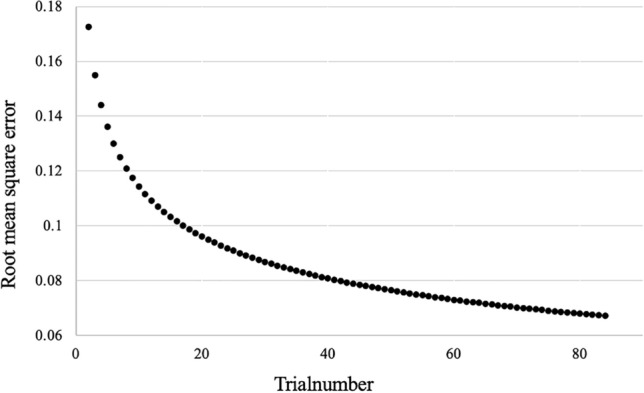
Root mean square error for our approximation of the probability distribution for the sum of up to 84 die reports. As a benchmark, we used exact probabilities computed in R 4.0.3 (R Core Team, 2018) using the dice package (v1.2; Arena, 2014). We did not make computations for higher numbers of die reports because computation for exact probabilities of 84 rolls already took excessive computation time. In our studies, the number of die reports was 90 (Experiment 1), 180 (Experiment 2) and 120 (Experiment 3)

**Fig. 7:**
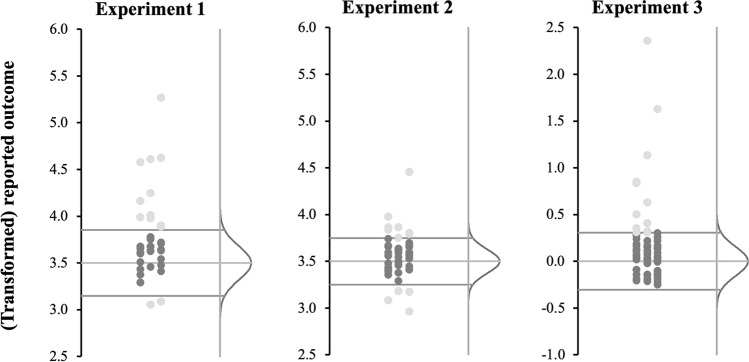
Distribution of (transformed) reported outcomes for all experiments. Each circle represents one participant. The curve at the right side of each plot shows the normal distribution of reported outcomes for this experiment. Non-outliers are shown in black, outliers (sum of reported outcomes exceeded the 97.5%-percentile or fell below the 2.5%-percentile of this normal distribution) are shown in light gray. The upper and lower horizontal lines represent the corresponding cut off values and the middle horizontal lines represent the expected values

## Data Availability

The datasets generated and analyzed during the current study are available in the OSF repository (Experiment 1–2: https://osf.io/bp8vt/; Experiment 3: https://osf.io/c9726/).

## Appendix 1: Descriptive data for recollection and die roll and report time

See Tables 1, 2, 3 and 4.

## Appendix 2: Reported outcomes and memory performance in Experiment 2

See Fig. 4 and Tables 5 and 6.

**Table 5:** Means (standard deviations in brackets) of reported outcomes for blocks, incentive structures and block halves in Experiment 2

Incentive	Block 1		Block 2	
	Block half 1	Block half 2	Block half 1	Block half 2
All trials paid	3.61 (0.44)	3.54 (0.48)	3.67 (0.55)	3.65 (0.49)
One trial paid	3.49 (0.31)	3.50 (0.35)	3.56 (0.37)	3.50 (0.38)

**Table 6:** Means (standard deviations in brackets) of memory performance for blocks, incentive structures and block halves in Experiment 2

Incentive	Block 1		Block 2	
	Block half 1	Block half 2	Block half 1	Block half 2
All trials paid	0.69 (0.14)	0.69 (0.13)	0.71 (0.15)	0.68 (0.14)
One trial paid	0.67 (0.16)	0.67 (0.14)	0.69 (0.15)	0.67 (0.16)

## Appendix 3: Transformed reported outcomes and memory performance in Experiment 3

See Fig. 5 and Table 7.

**Table 7:** Means (standard deviations in brackets) of memory performance for blocks within incentive structure, incentive structures and letter string lengths in Experiment 3

Incentive	Five letters		Eleven letters	
	Block 1	Block 2	Block 1	Block 2
Gain	0.85 (0.16)	0.85 (0.15)	0.55 (0.20)	0.59 (0.20)
Loss	0.87 (0.15)	0.86 (0.15)	0.60 (0.19)	0.61 (0.20)

## Appendix 4: Distribution of lying behavior within our samples

To analyze whether the share of outliers differed between cognitive load conditions, we pooled the data of all three studies for higher statistical power and calculated the sum of reported outcomes under high and low cognitive load for each participant (dropping the medium load conditions of Experiments 1 and 2). We classified participants as outliers when the cumulative probability for their sum of reported outcomes exceeded 90% (high outliers) or respectively fell below 10% (low outliers). These exact probabilities were computed in R (v4.0.3; R Core Team, 2020) using the dice package (v1.2; Arena, 2014). The full reproducible code is available in the OSF repository (https://osf.io/c9726/). Such a liberal outlier criterium was chosen here to circumvent floor effects and therefore increase statistical power in the following analyses. We calculated two separate McNemar-Tests (applying an Edwards correction) with outlier (yes vs. no) and cognitive load condition (high vs. low) for low and high outliers, respectively (see Tables 8 and 9 for the number of low and high outliers under both cognitive load conditions). Neither the share of low outliers (low load: 7.89%, high load: 7.24%), *p* > 0.999, nor the share of high outliers (low load: 28.29%, high load: 21.05%), *p* = 0.108, differed significantly between cognitive load conditions.

**Table 8:** Number of low outliers under low and high cognitive load

		Low outliers under high load		
		No	Yes	$\Sigma$
Low outliers under low load	No	130	10	140
	Yes	11	1	12
	$\Sigma$	141	11	152

**Table 9:** Number of high outliers under low and high cognitive load

		High outliers under high load		
		No	Yes	$\Sigma$
High outliers under low load	No	95	14	109
	Yes	25	18	43
	$\Sigma$	120	32	152

Next, we identified outliers across cognitive load conditions for each individual study (including also the medium cognitive load conditions in Experiments 1 and 2). Due to the high number of trials for the whole experimental design, a computation of exact probabilities for the sum of reported outcomes would require excessive computation power. Therefore, we instead assumed an approximative normal distribution (*μ*(*X*) = *E*(*X*) * *n*, *σ*(*X*) = $\sqrt{({n }* \frac{35}{12})}$; expected value E was 3.5 in Experiments 1 and 2, and 0 in Experiment 3)^4^ to approximate the probability of a given total sum X of reports for n die rolls.

As Fig. 6 shows, this normal distribution is a suitable approximation for a high number of die rolls when compared against exact probabilities.

For our analyses of die reports as a function of cognitive load conditions, we resorted to a more conservative definition of outliers than above. We defined high outliers as participants whose sum of reported outcomes across load conditions exceeded the 97.5%-percentile of the normal distribution of the specific experiment (Experiment 1: 27.5% of participants, Experiment 2: 17.4% of participants; Experiment 3: 18.0% of participants; see Fig. 7) and low outliers as participants whose sum of reported outcomes across load conditions was below the 2.5%-percentile (Experiment 1: 5.0% of participants, Experiment 2: 8.7% of participants; Experiment 3: no low outliers; see Fig. 7).

None of our hypotheses predicted low outliers. Further, such reporting behavior is odd, considering that it points to strategy use promoting dishonest reports that would result in less reward than a fully honest report. Therefore, we repeated our main analysis of lying behavior for Experiment 1 and Experiment 2 without these outliers. We only report main effects of letter string length and significant interactions with this factor here for brevity. In Experiment 1 (two letters: *M* = 3.87, SD = 0.50; five letters: *M* = 3.83, SD = 0.51; eight letters: *M* = 3.68, SD = 0.42), *F*(2, 74) = 4.79, *p* = 0.011, *η*_p_^2^ = 0.12, and in Experiment 2 (five letters: *M* = 3.65, SD = 0.34; eight letters: M = 3.63, SD = 0.22; eleven letters: *M* = 3.53, SD = 0.23), *F*(1.64, 67.13) = 4.03, *p* = 0.030, *η*_p_^2^ = 0.09, reported outcomes significantly declined when the number of letters increased.

We further excluded all high outliers and repeated our main analysis of lying behavior for each experiment to scrutinize whether the impact of cognitive load on lying behavior would be evident without prolific liars. After these data exclusions, in none of the experiments (transformed) reported outcomes were affected by letter string condition (Experiment 1: two letters: *M* = 3.64, SD = 0.29; five letters: *M* = 3.60, SD = 0.26; eight letters: *M* = 3.54, SD = 0.27, *F* < 1; Experiment 2: five letters: *M* = 3.55, SD = 0.23; eight letters: M = 3.57, SD = 0.16; eleven letters: *M* = 3.47, SD = 0.15), *F*(2, 66) = 2.89, *p* = 0.063, *η*_p_^2^ = 0.08; Experiment 3: five letters: *M* = 0.01, SD = 0.24; eleven letters: *M* = 0.04, SD = 0.23), *F* < 1). In Experiment 3, letter string condition and incentive structure interacted significantly, *F*(1, 53) = 6.49, *p* = 0.014, *η*_p_^2^ = 0.11. While we did not observe a significant effect of letter string condition on transformed reported outcomes in the gain condition of Experiment 3 (five letters: *M* = 0.11, SD = 0.41; eleven letters: *M* = − 0.01, SD = 0.39),* t*(53) = 1.75, *p* = 0.086, *d*_*z*_ = 0.24, in the loss condition, transformed reported outcomes significantly increased when the number of letters increased (five letters: *M* = − 0.09, SD = 0.48; eleven letters: *M* = 0.08, SD = 0.40),* t*(53) = 2.12, *p* = 0.039, *d*_*z*_ = 0.29.

## Footnotes

1 The post-questionnaire showed that one participant correctly guessed our hypotheses but as we did not preregister data exclusions, the following calculations are based on the whole sample. Excluding the corresponding dataset did not change any statistical decision.

2 Due to an uneven number of trials per block we compared trials 1–22 against trials 24–45 in each block.

3 We thank Jonathan Baron for his in-depth exploration of our data that encouraged us to conduct these analyses.

## References

[CR1] Arena, D. (2014). Dice: Calculate probabilities of various dice-rolling events. R package version 1.2. Retrieved 19 Dec 2021, from https://CRAN.R-project.org/package=dice.

[CR2] Benjamin DJ, Brown SA, Shapiro JM (2013). Who is “behavioral”? Cognitive ability and anomalous preferences. Journal of the European Economic Association.

[CR3] Bereby-Meyer Y, Shalvi S (2015). Deliberate honesty. Current Opinion in Psychology.

[CR4] Bogliacino F, Montealegre F (2020). Do negative economic shocks affect cognitive function, adherence to social norms and loss aversion?. Journal of the Economic Science Association.

[CR5] Cameron, J. S., Miller, D. T., & Monin, B. (2010). Deservingness and unethical behavior in loss and gain frames. In *Academy of Management annual meeting* (No. 12987).

[CR6] Capraro V (2017). Does the truth come naturally? Time pressure increases honesty in one-shot deception games. Economics Letters.

[CR7] Capraro V, Schulz J, Rand DG (2019). Time pressure and honesty in a deception game. Journal of Behavioral and Experimental Economics.

[CR8] Debey E, De Houwer J, Verschuere B (2014). Lying relies on the truth. Cognition.

[CR9] Deck C, Jahedi S (2015). The effect of cognitive load on economic decision making: A survey and new experiments. European Economic Review.

[CR10] Dickinson DL, McEvoy DM (2021). Further from the truth: The impact of moving from in-person to online settings on dishonest behavior. Journal of Behavioral and Experimental Economics.

[CR11] Druckman JN (2001). Evaluating framing effects. Journal of Economic Psychology.

[CR12] Du Y, Ma W, Sun Q, Sai L (2021). Collaborative settings increase dishonesty. Frontiers in Psychology.

[CR13] Duffy S, Smith J (2014). Cognitive load in the multi-player prisoner’s dilemma game: Are there brains in games?. Behavioral and Experimental Economics.

[CR14] Duran ND, Dale R, McNamara DS (2010). The action dynamics of overcoming the truth. Psychonomic Bulletin and Review.

[CR15] Farinha, A. C., & Maia, T. V. (2021). People exert more effort to avoid losses than to obtain gains. *Journal of Experimental Psychology: General.* Advance Online Publication. 10.1037/xge0001021. 10.1037/xge0001021 33734775

[CR16] Fischbacher U, Föllmi-Heusi F (2013). Lies in disguise—An experimental study on cheating. Journal of the European Economic Association.

[CR17] Foerster A, Pfister R, Schmidts C, Dignath D, Kunde W (2013). Honesty saves time (and justifications). Frontiers in Psychology.

[CR18] Foerster A, Wirth R, Berghoefer FL, Kunde W, Pfister R (2019). Capacity limitations of dishonesty. Journal of Experimental Psychology: General.

[CR19] Foerster A, Wirth R, Kunde W, Pfister R (2017). The dishonest mind set in sequence. Psychological Research.

[CR20] Furedy JJ, Davis C, Gurevich M (1988). Differentiation of deception as a physiological process: A psychophysiological approach. Psychophysiology.

[CR21] Gächter, S., Johnson, E., & Herrmann, A. (2010). Individual-level loss aversion in riskless and risky choices. In *CeDEx discussion paper series* (No. 20). Retrieved December 12, 2021, from http://hdl.handle.net/10419/49656.

[CR22] Gamer M, Rill HG, Vossel G, Gödert HW (2006). Psychophysiological and vocal measures in the detection of guilty knowledge. International Journal of Psychophysiology.

[CR23] Garrett N, Lazzaro SC, Ariely D, Sharot T (2016). The brain adapts to dishonesty. Nature Neuroscience.

[CR24] Gawronski B, Creighton LA, Carlston DE (2013). Dual process theories. The Oxford handbook of social cognition.

[CR25] Gilbert DT, Hixon JG (1991). The trouble of thinking: Activation and application of stereotypic beliefs. Journal of Personality and Social Psychology.

[CR26] Gneezy, U., Kajackaite, A., & Sobel, J. (2018). Lying aversion and the size of the lie. *The American Economic Review*, *108*(2), 419–453. https://jstor.org/stable/10.2307/26527910.

[CR27] Grolleau G, Kocher MG, Sutan A (2016). Cheating and loss aversion: Do people cheat more to avoid a loss?. Management Science.

[CR28] Guillemette MA, Russel NJ, Larsen J (2014). Loss aversion under cognitive load. Journal of Personal Finance.

[CR29] Harinck F, Van Dijk E, Van Beest I, Mersmann P (2007). When gains loom larger than losses: Reversed loss aversion for small amounts of money. Psychological Science.

[CR30] Hilbig BE, Hessler CM (2013). What lies beneath: How the distance between truth and lie drives dishonesty. Journal of Experimental Social Psychology.

[CR31] Kahneman D, Tversky A (1979). Prospect theory: An analysis of decision under risk. Econometrica.

[CR32] Kern MC, Chugh D (2009). Bounded ethicality: The perils of loss framing. Psychological Science.

[CR33] Köbberling V, Wakker PP (2005). An index of loss aversion. Journal of Economic Theory.

[CR34] Köbis NC, Verschuere B, Bereby-Meyer Y, Rand D, Shalvi S (2019). Intuitive honesty versus dishonesty: Meta-analytic evidence. Perspectives on Psychological Science.

[CR35] Kouchaki M, Smith-Crowe K, Brief AP, Sousa C (2013). Seeing green: Mere exposure to money triggers a business decision frame and unethical outcomes. Organizational Behavior and Human Decision Processes.

[CR36] Kristal AS, Whillans AV, Bazerman MH, Gino F, Shu LL, Mazar N, Ariely D (2020). Signing at the beginning versus at the end does not decrease dishonesty. Proceedings of the National Academy of Sciences.

[CR37] Kröll, M., & Rustagi, D. (2016). Shades of dishonesty and cheating in informal milk markets in India. In *SAFE working papers* (No. 134). Retrieved December 7, 2021, from https://core.ac.uk/display/43279994.

[CR38] Lohse T, Simon SA, Konrad KA (2018). Deception under time pressure: Conscious decision or a problem of awareness?. Journal of Economic Behavior and Organization.

[CR39] Mazar N, Amir O, Ariely D (2008). The dishonesty of honest people: A theory of self-concept maintenance. Journal of Marketing Research.

[CR40] Mitra A, Shahriar Q (2020). Why is dishonesty difficult to mitigate? The interaction between descriptive norm and monetary incentive. Journal of Economic Psychology.

[CR41] Norton MI, Mochon D, Ariely D (2012). The IKEA effect: When labor leads to love. Journal of Consumer Psychology.

[CR42] Nuñez JM, Casey BJ, Egner T, Hare T, Hirsch J (2005). Intentional false responding shares neural substrates with response conflict and cognitive control. NeuroImage.

[CR43] Pfattheicher S, Schindler S, Nockur L (2019). On the impact of Honesty-Humility and a cue of being watched on cheating behavior. Journal of Economic Psychology.

[CR44] Pfister, R. (2022). Operationalization and generalization in experimental psychology: A plea for bold claims. In D. Gozli & J. Valsiner (Eds.), *Experimental psychology: Ambitions and possiblities* (pp. 1–32). Preprint: 10.31234/osf.io/k38y5.

[CR45] Pfister R, Janczyk M (2013). Confidence intervals for two sample means: Calculation, interpretation, and a few simple rules. Advances in Cognitive Psychology.

[CR46] R Core Team (2020). R: A language and environment for statistical computing. R foundation for statistical computing, Vienna, Austria. Retrieved November 29, 2021, from https://www.R-project.org.

[CR47] Schindler S, Pfattheicher S (2017). The frame of the game: Loss-framing increases dishonest behavior. Journal of Experimental Social Psychology.

[CR48] Serota KB, Levine TR (2015). A few prolific liars: Variation in the prevalence of lying. Journal of Language and Social Psychology.

[CR49] Serota KB, Levine TR, Boster FJ (2010). The prevalence of lying in America: Three studies of self-reported lies. Human Communication Research.

[CR50] Shipstead Z, Engle RW (2013). Interference within the focus of attention: Working memory tasks reflect more than temporary maintenance. Journal of Experimental Psychology: Learning, Memory, and Cognition.

[CR51] Spence SA, Farrow TFD, Herford AE, Wilkinson ID, Zheng Y, Woodruff PWR (2001). Behavioural and functional anatomical correlates of deception in humans. NeuroReport.

[CR52] Sporer SL (2016). Deception and cognitive load: Expanding our horizon with a working memory model. Frontiers in Psychology.

[CR53] Ting C (2018). The feedback loop of rule-breaking: Experimental evidence. The Social Science Journal.

[CR54] Tversky A, Kahneman D (1981). The framing of decisions and the psychology of choice. Science.

[CR55] Van der Cruyssen I, D’hondt J, Meijer E, Verschuere B (2020). Does honesty require time? Two preregistered direct replications of experiment 2 of Shalvi, Eldar, and Bereby-Meyer (2012). Psychological Science.

[CR56] Van’t Veer AE, Stel M, van Beest I (2014). Limited capacity to lie: Cognitive load interferes with being dishonest. Judgment and Decision Making.

[CR57] Verschuere B, Meijer EH, Jim A, Hoogesteyn K, Orthey R, McCarthy RJ (2018). Registered replication report on Mazar, Amir, and Ariely (2008). Advances in Methods and Practices in Psychological Science.

[CR58] Vrij A, Fisher RP, Blank H (2017). A cognitive approach to lie detection: A meta-analysis. Legal and Criminological Psychology.

[CR59] Walczyk JJ, Igou FP, Dixon AP, Tcholakian T (2013). Advancing lie detection by inducing cognitive load on liars: A review of relevant theories and techniques guided by lessons from polygraph-based approaches. Frontiers in Psychology.

[CR60] Welsh DT, Ordonez LD, Snyder DG, Christian MS (2015). The slippery slope: How small ethical transgressions pave the way for larger future transgressions. Journal of Applied Psychology.

